# Современное состояние исследований в области ожирения: генетические аспекты, роль микробиома и предрасположенность к COVID-19

**DOI:** 10.14341/probl12775

**Published:** 2021-08-02

**Authors:** Я. Р. Тимашева, Ж. Р. Балхиярова, О. В. Кочетова

**Affiliations:** Институт биохимии и генетики Уфимского федерального исследовательского центра Российской академии наук; Башкирский государственный медицинский университет; Институт биохимии и генетики Уфимского федерального исследовательского центра Российской академии наук; Башкирский государственный медицинский университет; Университет Суррея; Институт биохимии и генетики Уфимского федерального исследовательского центра Российской академии наук

**Keywords:** ожирение, морбидное ожирение, бариатрическая хирургия, кишечный микробиом, COVID-19

## Abstract

Число людей с ожирением в мире достигло 700 млн человек и продолжает неуклонно увеличиваться. Проблема приобретает особую актуальность в связи с повышенным риском осложненного течения и смертности от COVID-19 у пациентов с ожирением. Увеличение распространенности ожирения, в том числе морбидного, связывают с действием внешних и поведенческих факторов, что приводит к стигматизации людей с ожирением, поскольку их проблемы считают обусловленными неправильным образом жизни, характером питания и другими управляемыми факторами. Тем не менее установлено существование наследственной предрасположенности к ожирению, которая носит выраженный полигенный характер. К развитию морбидного ожирения могут приводить редкие мутации, оказывающие значительный эффект на энергетический обмен и отложение жира, однако у большинства пациентов они не выявляются. Низкое разнообразие генов микробиома коррелирует с метаболическими нарушениями (хроническим воспалением, инсулинорезистентностью, размером адипоцитов), а также успешностью оперативных вмешательств, направленных на коррекцию веса (бариатрической хирургии), но данных об отдаленных последствиях бариатрической хирургии и изменении состава, генетического разнообразия и активности микробиома до и после хирургического вмешательства пока недостаточно. В обзоре представлены результаты исследований генетических особенностей пациентов, страдающих ожирением, молекулярных механизмов патогенеза ожирения, способствующих неблагоприятному течению коронавирусной инфекции, а также эволюции микробиома пациентов при бариатрической хирургии, проливающие свет на природу развития заболевания и создающие предпосылки для определения потенциальных мишеней для лекарственной терапии и разработки персонализированных эффективных подходов в диагностике, лечении и профилактике ожирения.

## Abstract (en)

Obesity affects over 700 million people worldwide and its prevalence keeps growing steadily. The problem is particularly relevant due to the increased risk of COVID-19 complications and mortality in obese patients. Obesity prevalence increase is often associated with the influence of environmental and behavioural factors, leading to stigmatization of people with obesity due to beliefs that their problems are caused by poor lifestyle choices. However, hereditary predisposition to obesity has been established, likely polygenic in nature. Morbid obesity can result from rare mutations having a significant effect on energy metabolism and fat deposition, but the majority of patients does not present with monogenic forms. Microbiome low diversity significantly correlates with metabolic disorders (inflammation, insulin resistance), and the success of weight loss (bariatric) surgery. However, data on the long-term consequences of bariatric surgery and changes in the microbiome composition and genetic diversity before and after surgery are currently lacking. In this review, we summarize the results of studies of the genetic characteristics of obesity patients, molecular mechanisms of obesity, contributing to the unfavourable course of coronavirus infection, and the evolution of their microbiome during bariatric surgery, elucidating the mechanisms of disease development and creating opportunities to identify potential new treatment targets and design effective personalized approaches for the diagnosis, management, and prevention of obesity.

## ВВЕДЕНИЕ

Согласно определению Всемирной организации здравоохранения, ожирение — это патологическое накопление жира, представляющее риск для здоровья [1]. Для диагностики ожирения используется индекс массы тела (ИМТ), рассчитываемый как отношение массы тела в килограммах к квадрату роста в метрах. Нормальные показатели ИМТ находятся в пределах 18,5–24,9 кг/м2, ИМТ ≥25 кг/м2 классифицируется как наличие излишней массы тела, ИМТ ≥30 кг/м2 — ожирение, а ИМТ ≥40 кг/м2 (либо ИМТ ≥35 кг/м2 при наличии серьезных осложнений) — морбидное ожирение. В России распространенность ожирения составляет 27,5% среди мужчин и 31,4% среди женщин [2]. Отмечается рост числа людей, страдающих ожирением; в США доля лиц с морбидным ожирением выросла с 4,7% в 1999–2000 гг. до 9,2% в 2017–2018 гг., а в 1990 г. составляла лишь 0,9% [3]. Ожирение значительно снижает ожидаемую продолжительность жизни, способствуя развитию кардиометаболических нарушений (сахарный диабет 2 типа (СД2), дислипидемии, ишемическая болезнь сердца, инсульт, артериальная гипертензия) и неметаболических заболеваний (гастроэзофагально-рефлюксная болезнь, неалкогольный стеатогепатит, цирроз печени, рак, нарушения сна, депрессия и поражение опорно-двигательного аппарата) [4].

Ожирение — это многофакторное заболевание, возникающее в результате положительного баланса энергии, когда количество энергии, обеспечиваемое потребляемой пищей, превышает затраты энергии в ходе жизнедеятельности. Избыток энергии откладывается в жировых депо в виде триглицеридов, приводя к появлению избыточной массы тела, а впоследствии — к развитию ожирения [5]. Одними из основных факторов развития ожирения принято считать изменившийся характер питания и преобладание в рационе полуфабрикатов и фастфуда, содержащих большое количество энергии, а также снижение физической активности как часть современного образа жизни [6]. Социальные факторы также вносят вклад в развитие ожирения: если в странах с низким уровнем дохода ожирением страдают, как правило, состоятельные городские жители среднего возраста, преимущественно женщины, то в странах с высоким уровнем дохода ожирение распространено среди мужчин и женщин всех возрастов, но при этом больные, как правило, относятся к социально уязвимым группам населения [7].

Несмотря на значимость влияния факторов внешней среды, нельзя недооценивать роль генетического компонента в развитии ожирения. В ходе ранних семейных [8–11] и близнецовых [12–14] исследований было установлено, что вклад генетических факторов в вариабельность ИМТ может достигать 70–80%. Популяционные исследования распространенности ожирения в различных этнических группах также подчеркивают влияние генетических особенностей [15]. В то время как в ряде случаев ожирение является составной частью различных хромосомных синдромов и результатом влияния мутаций отдельных генов (рис. 1), по большей части, по-видимому, развитие ожирения обусловлено сложным взаимодействием внешних факторов, способствующих ожирению (“obesogenic environment”) и индивидуальной наследственной предрасположенности [16][17].

**Figure:**
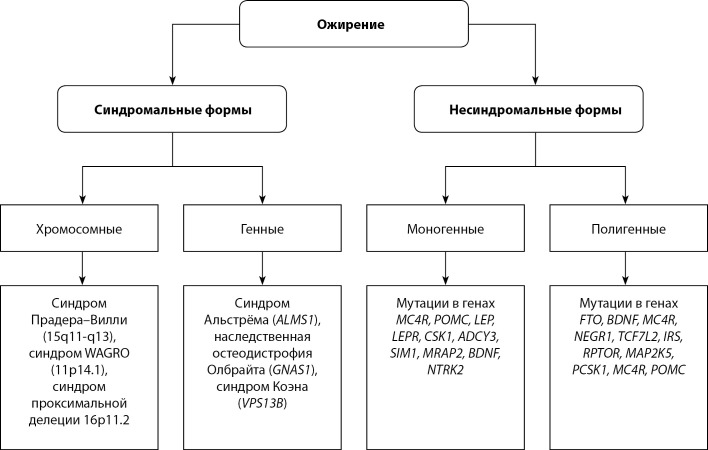
Рисунок 1. Генетическая структура ожирения.

## СИНДРОМАЛЬНЫЕ ФОРМЫ ОЖИРЕНИЯ

К настоящему времени в литературе описаны 79 различных синдромов, включающих ожирение в качестве одного из клинических признаков, из них 30 — с установленной генетической причиной развития (19 — полностью, 11 — частично), 27 — синдромы, развитие которых связывают с мутациями в определенном хромосомном участке, и 22 — синдромы, для которых пока не найдены ни ген(ы), ни хромосомный участок, отвечающие за их развитие [18]. Наиболее полно охарактеризованные хромосомные наследственные синдромы, связанные с развитием ожирения, представлены в таблице 1. К ним относится синдром Барде–Бидля, который, как было показано к настоящему моменту, может быть связан с дефектами 16 различных хромосом, сопровождающимися нарушениями структуры 21 гена, тогда как примерно в 25% случаев генетическую причину заболевания установить не удается [19]. Наиболее распространенной формой ожирения, обусловленного хромосомными нарушениями, является синдром Прадера–Вилли (15q11-13). Описан Прадера–Вилли-подобный фенотип, который может наблюдаться при делециях участков хромосомы 6, включающих гены SIM1 или MRAP2, и при дупликации участка 6q16.3q23.3 с образованием сверхчисленной маркерной хромосомы (sSMC) [20][21]. Сообщается также о пациентах с синдромом ломкой Х-хромосомы (fragile X-syndrome — FXS) с Прадера–Вилли фенотипом (Prader–Willi phenotype — PWP), у которых наблюдались возникающая в детстве гиперфагия с отсутствием чувства насыщения и ожирение, а также более выраженные, чем при FXS без PWP, поведенческие расстройства и социальная дезадаптация [22].

**Table:** Таблица 1. Хромосомные синдромы, одним из клинических признаков которых является ожирение

СиндромБарде–Бидля (Лоуренса–Муна–Барде–Бидля)	11q13.2	BBS1	1:125 000–175 000	Пигментный ретинит, ожирение, нарушение функции почек, полидактилия, поведенческие расстройства, гипогонадизм
16q13	BBS2
3q11.2	BBS3 (ARL6)
15q24.1	BBS4
2q31.1	BBS5
20p12.2	BBS6 (MKKS)
4q27	BBS7
14q31.3	BBS8 (TTC8)
7p14.3	BBS9 (PTHB1)
12q21.2	BBS10
9q33.1	BBS11 (TRIM32)
4q27	BBS12
17q22	BBS13 (MKS1)
12q21.32	BBS14 (CEP290)
2p15	BBS15 (WDPCP)
1q43–q44	BBS16 (SDCCAG8)
3p21.31	BBS17 (LZTFL1)
10q25.2	BBS18 (BBIP1)
22q12.3	BBS19 (IFT27)
9p21.2	BBS20 (IFT172)
8q22.1	BBS21 (C8ORF37)
СиндромПрадера–Вилли	15q11.2	MKRN3	1 :15 000–30 000	Плохая прибавка в весе (failure to thrive) и трудности с кормлением в младенчестве, ожирение и гиперфагия в детстве, мышечная гипотония, гипоплазия гениталий, задержка развития, дефицит гормона роста, низкий рост, маленькие руки и ноги, поведенческие проблемы
15q11.2	MAGEL2
15q11.2	NDN
15q11.2	NPAP1
15q11.2	SNURF‐SNRPN
15q11.2	SNORD11, SNORD116
15q11–q13	Нарушения импринтинга
Прадера–Вилли-подобный синдром	6q16.3	SIM1	Не установлена	Задержка психомоторного развития, ожирение, гипотония и короткие конечности
6q14.2	MRAP2
6q16.3q23.3		1 случай
Xq27.3	FMR1	27 случаев	Ожирение, поведенческие нарушения, умственная отсталость
Наследственная остеодистрофия Олбрайта	20q13.32	GNAS	Не установлена	Брахиметафалангизм (укорочение метакарпальных и метатарзальных костей), низкий рост, ожирение и умственная отсталость
Синдром Альстрёма	2p13.1	ALMS1	Менее 1:1 000 000	Слепота, нарушение слуха, детское ожирение, инсулинорезистентность и сахарный диабет 2 типа
Синдром Карпентера	6p12.1–p11.2	RAB23	Около 40 случаев	Акроцефалия, синдактилия мягких тканей, брахи- или агенезия мезофаланги кистей и стоп, преаксиальная полидактилия, врожденные пороки сердца, умственная отсталость, гипогенитализм, ожирение и пупочная грыжа
Синдром CHOPS	5q31.1	AFF4	Менее 1:1 000 000	Когнитивные нарушения, грубые черты лица, пороки сердца, ожирение, поражение легких, низкий рост и дисплазия скелета
СиндромЧадли– Лоури	Xq21.1	ATRX	Менее 1:1 000 000	Умственная отсталость, низкий рост, ожирение, гипогонадизм и характерные изменения лицевого скелета
Синдром Коэна	8q22.2	VPS13B	Около 200 случаев	Умственная отсталость, лицевой дисморфизм, микроцефалия, дистрофия сетчатки, абдоминальное ожирение, гипермобильность суставов и перемежающаяся нейтропения
Синдром Ларона	5p13.1–p12	GHR	Около 250 случаев	Низкий рост, снижение мышечной силы и выносливости, ожирение, гипогликемия в младенчестве, гипогенитализм, задержка полового созревания, тонкие и ломкие волосы и аномалии зубов
Синдром MORM	9q34.3	INPP5E	14 членов одной семьи	Умеренная умственная отсталость, абдоминальное ожирение, врожденная непрогрессирующая дистрофия сетчатки и микропенис
СиндромСмит–Магенис	17p11.2	RAI1	1:15 000–25 000	Умственная отсталость, задержка развития речевых навыков, характерный лицевой фенотип, нарушения сна, ожирение и поведенческие проблемы
Синдром WAGRO	11p14.1	BDNF	Не установлена	Аниридия, опухоль Вильмса, аномалии мочеполовой системы, ожирение и рост, а также умственная отсталость
Синдром проксимальной делеции 16p11.2	16p11.2	SH2B1	Не установлена	Ожирение, аутизм, умственная отсталость, врожденные аномалии и задержка развития
16p11.2	KCTD13

 

Кроме того, ожирение является одним из клинических проявлений таких синдромов, как псевдогипопаратиреоз Ia типа (наследственная остеодистрофия Олбрайта), синдром CHOPS (Cognitive impairment/Coarse facial features, Heart defects, Obesity, Pulmonary problems, Short stature / Skeletal abnormalities), синдром MORM (Mental retardation, truncal Obesity, Retinal dystrophy and Micropenis) и синдром WAGRO (Wilms tumor, Aniridia, Genitourinary anomalies, mental Retardation, Obesity) [23].

К редким формам синдромального ожирения можно отнести синдром Ларона (дефицит рецепторов соматотропного гормона), синдром Карпентера (акроцефалополисиндактилия II типа) и синдром Коэна, частота встречаемости которого повышена в финской популяции, а также описаны семейные случаи у амишей, ирландцев, греков и некоторых других представителей средиземноморских стран.

## МОНОГЕННЫЕ ФОРМЫ ОЖИРЕНИЯ

К настоящему времени развитие моногенных форм ожирения связывают с несколькими генами (MC4R, LEP, LEPR, PCSK1, ADCY3, POMC, MRAP2), продукты которых входят в состав лептин-меланокортинового пути, участвующего в регуляции энергетического обмена [24][25]. Несмотря на то что моногенные формы ожирения встречаются чрезвычайно редко, описаны гомозиготные мутации в этих генах, обладающие полной пенетрантностью и приводящие к развитию заболевания (табл. 2). Наряду с этим выделяют так называемые олигогенные формы ожирения у взрослых и детей, которые связаны с гетерозиготными мутациями в этих же генах и характеризуются различной степенью тяжести в зависимости от влияния средовых факторов [26][27].

**Table:** Таблица 2. Моногенные формы ожирения

Ген	Хромосомная локализация	Продукт	Клинические проявления
MC4R	18q21.32	Рецептор меланокортина 4	Морбидное ожирение с ранним началом, гиперфагия, гиперинсулинемия, увеличение безжировой массы тела, повышение минеральной плотности костей
POMC	2p23.3	Проопиомеланокортин	Ожирение тяжелой степени с манифестацией в раннем возрасте, гиперфагия, недостаточность адренокортикотропного гормона, гипотиреоз, рыжий цвет волос
LEP	7q32.1	Лептин	Морбидное ожирение с ранним началом, дефицит гонадотропного и тиреотропного гормонов, иммунные нарушения
LEPR	1p31.3	Рецептор лептина	Морбидное ожирение с ранним началом, дефицит гонадотропного, соматотропного и тиреотропного гормонов, иммунные нарушения
ADCY3	2p23.3	Аденилатциклаза 3	Ожирение (абдоминальное), инсулинорезистентность, дислипидемия, диабет 2 типа, нарушения обоняния
BDNF	11p14.1	Нейротрофический фактор мозга	Морбидное ожирение, гиперфагия, гиперактивность, когнитивные расстройства, нарушения памяти и болевой чувствительности
NTRK2	9q21.33	Нейротрофический рецептор тирозинкиназы 2	Морбидное ожирение с ранним началом, задержка развития, стереотипное поведение, нарушения памяти и обучения, сниженная болевая чувствительность
MRAP2	6q14.2	Вспомогательный белок 2 рецептора меланокортина 2	Ожирение различной степени, гиперфагия, метаболический синдром (артериальная гипертензия, гипергликемия)
PCSK1	5q15	Пропротеин-конвертаза субтилизина/кексина типа 1	Морбидное ожирение с ранним началом, недостаточность гонадотропного, соматотропного, тиреотропного гормонов, постпрандиальный гипогликемический синдром, центральный несахарный диабет, хроническая тяжелая диарея с мальабсорбцией, возникающая в неонатальном периоде
SIM1	6q16.3	Целенаправленный гомолог-1	Морбидное ожирение с ранним началом, задержка развития, гипотония, эмоциональная лабильность, аутистическое поведение

Выявление мутаций в гене лептина LEP, связанных с развитием выраженного ожирения и сахарного диабета 2 типа у мышей, а также с морбидным ожирением у детей, положило начало обнаружению редких мутаций, приводящих к развитию ожирения [28]. Лептин регулирует энергетический баланс, действуя через меланокортин-зависимый и меланокортин-независимый пути [29]. Благодаря связыванию лептина со своими рецепторами в гипоталамусе, центральная нервная система получает сигналы от жировой ткани о том, что в организме достаточно запасов энергии. В гипоталамусе находится центр, осуществляющий долгосрочную регуляцию энергетического обмена путем интеграции сигналов периферического и центрального происхождения об энергетическом и нутрициональном статусе организма и состоянии внешней среды. Мутации в гене лептина приводят к гиперфагии (перееданию), сниженной двигательной активности, снижению тонуса симпатической нервной системы, гипофункции щитовидной железы, гипогонадизму и нарушению Т-клеточного иммунитета. Есть сообщения о том, что терапия рекомбинантным лептином благоприятно сказывалась на состоянии пациентов с морбидным ожирением, сахарным диабетом и гипогонадизмом, обусловленными дефицитом лептина [30].

На молекулярном уровне действие лептина проявляется стимуляцией выработки проопиомеланокортина (POMC) в нейронах аркуатного и дорсомедиального ядер гипоталамуса. POMC является предшественником ряда пептидных гормонов, в том числе альфа-меланоцитстимулирующего гормона (α-MSH), или альфа-меланотропина, который, связываясь с рецептором меланокортина 4 (MC4R), подавляет аппетит. Было обнаружено, что мутации в генах POMC и MC4R связаны с развитием морбидного ожирения [31][32]. Следует отметить, что гетерозиготные мутации в гене MC4R выявляются у 5% пациентов с ожирением, развивающимся в детском возрасте; таким образом, дефекты гена MC4R являются наиболее частой причиной моногенного ожирения [33]. При мутациях гена POMC, связанных с дефицитом пептида, могут также возникать нарушения пигментного обмена вследствие недостаточности альфа-меланотропина, что обусловливает характерную рыжую окраску волос пациентов [32].

Дифференциальное расщепление POMC, которое может приводить к образованию α-MSH, а также адренокортикотропного гормона и бета-эндорфина, осуществляется с помощью пропротеинконвертазы-1 (PCSK1), которая также участвует в процессинге проинсулина и проглюкагона в поджелудочной железе [34]. У гомозигот и компаунд-гетерозигот по ряду мутаций в гене PCSK1 может нарушаться процессинг POMC, что проявляется в развитии ожирения, глюкокортикоидной недостаточности, гипогонадотропного гипогонадизма, постпрандиальной гипогликемии и мальабсорбции [35][36].

Ген ADCY3 кодирует аденилатциклазу, расположенную в первичных ресничках, в частности в гипоталамусе. Мутации в гене приводят к развитию ожирения вследствие нарушения передачи сигнала циклического АМФ (цАМФ) в пути, инициированном MC4R. Характерной особенностью моногенных форм ожирения, обусловленных дефектом ADCY3, являются сопутствующие им нарушения обоняния (аносмия) [37][38].

Ген SIM1 кодирует фактор транскрипции, участвующий в сигналинге MC4R и предположительно задействованный в формировании супраоптического и паравентрикулярного ядер гипоталамуса [20][39][40]. Гетерозиготные мутации гена связаны с развитием морбидных форм ожирения, иногда сопровождающихся снижением интеллектуального уровня [20][39].

Ген MRAP2 кодирует вспомогательный трансмембранный протеин, который связывается с рецепторами, сопряженными с G-белком, и тем самым регулирует передачу обеспечиваемого ими сигнала, в частности, стимулирует выработку цАМФ, индуцированную MC4R, в ответ на αMSH [41][42]. К настоящему времени идентифицировано более 20 мутаций в гене MRAP2, связанных с развитием ожирения [43]. В отличие от других моногенных форм, ожирение, вызванное дефицитом MRAP2, как правило, не является морбидным, но сопровождается развитием гипергликемии и/или артериальной гипертензии [43]. Это связывают с тем, что, в отличие от других генов лептин-меланокортинового пути, MRAP2 экспрессируется в других органах и тканях, помимо головного мозга, в частности бета-клетках островков Лангерганса поджелудочной железы, и может влиять на их функцию, снижая секрецию инсулина [44].

Описаны также случаи моногенного ожирения, обусловленные мутациями в гене нейротрофического рецептора тирозинкиназы 2 (NTRK2) и его лиганда нейротрофического фактора мозга (BDNF), характерной особенностью которых являются когнитивные расстройства, нарушения памяти, обучения и болевой чувствительности, предположительно, вследствие измененной функции гипоталамуса [45][46].

## ПОЛИГЕННЫЕ ФОРМЫ ОЖИРЕНИЯ

Несмотря на то что к настоящему моменту выявлено значительное количество моногенных форм, у большинства пациентов ожирение представляет собой многофакторное заболевание, обусловленное сочетанным влиянием факторов внешней среды и генетических факторов. С помощью полногеномных ассоциативных исследований (GWAS) было идентифицировано более 200 полиморфных вариантов, ассоциированных с ожирением (https://www.ebi.ac.uk/gwas/efotraits/EFO_0001073), и более 4200, ассоциированных с ИМТ (https://www.ebi.ac.uk/gwas/efotraits/EFO_0004340). Тем не менее вместе эти локусы объясняют лишь 5% индивидуальной вариабельности ИМТ [47]. Наследственная предрасположенность к ожирению может также проявляться опосредованно, в виде измененных поведенческих реакций на средовые факторы, или ген-средовых взаимодействий [48]. В частности, особенности питания, физическая активность, социоэкономический статус могут модифицировать степень проявления генетической предрасположенности к ожирению [49]. Несмотря на то что урбанизированный образ жизни современного человека, несомненно, повлиял на развитие эпидемии ожирения, нельзя упускать из виду то обстоятельство, что эволюционно сложившиеся молекулярные механизмы обмена направлены на накопление и депонирование запасов энергии и, следовательно, также способствуют развитию ожирения. Предполагается, что такие негенетические факторы, как характер питания, физические упражнения или хирургические вмешательства, направленные на снижение веса, индуцируют динамические изменения эпигенетических паттернов, модулируя тем самым активность генов [16]. Были обнаружены значимые корреляции клинических параметров, характерных для ожирения, и эпигенетических паттернов, обнаруживаемых в клетках крови, жировой ткани, печени и скелетной мускулатуры [50–53].

Анализ результатов GWAS показал, что локусы, ассоциированные с ожирением, включают гены, продукты которых участвуют в контроле аппетита и чувства насыщения (MC4R, ген нейротрофического фактора головного мозга — BDNF, ген регулятора роста нейронов 1 — NEGR1), выработки инсулина (ген фактора транскрипции 7, подобного второму, — TCF7L2, ген субстрата 1 инсулинового рецептора — IRS1), образования клеток жировой ткани, а также энергетического и липидного метаболизма (ген, ассоциированный с жировой массой и ожирением, — FTO; ген регуляторного белка, связанного с mTOR, — RPTOR; ген митоген-активируемой киназы 5 — MAP2K5). Кроме того, анализ генов, ассоциированных с кардиометаболическими заболеваниями, имеющими общий патогенез (ожирение, диабет, артериальная гипертензия, ишемическая болезнь сердца, рак молочной железы, синдром поликистозных яичников, рак почки), позволил выявить локусы и молекулярные пути, являющиеся общими для этих заболеваний [54]. Также следует отметить, что вдобавок к редким мутациям потери функции (loss-of-function) в генах PCSK1, MC4R и POMC также обнаруживаются полиморфные варианты, ассоциированные с полигенными формами ожирения в различных популяциях [55–58].

Безусловно, GWAS являются мощным инструментом для анализа генетической архитектуры сложных признаков, таких как ожирение, но интерпретация результатов GWAS затруднена. Большинство генетических вариантов, ассоциированных с ожирением, локализовано в некодирующих участках генома, в которых могут находиться регуляторные элементы, играющие важную роль в контроле транскрипционной активности генов, причем зачастую расположенные вдали от генетического варианта, для которого обнаружена ассоциация с ожирением. Поэтому, основываясь только на информации о расположении того или иного полиморфного варианта вблизи какого-либо гена, невозможно сделать вывод о том, что различные аллели этого полиморфизма способны изменять функцию этого гена. В частности, долгое время оставался неясным молекулярный механизм, лежащий в основе выявленных ассоциаций полиморфных вариантов в гене FTO с ожирением, пока не было установлено, что полиморфный вариант rs1421085, находящийся в интроне гена FTO и ассоциированный с ИМТ, связан с нарушением последовательности репрессора гена белка 4A, содержащего AT-богатый интерактивный домен (ARID5B), который регулирует экспрессию генов из семейства ирокез-гомеобокс IRX3 и IRX5 [59][60]. Это приводит к нарушению термогенеза, увеличению размеров жировых клеток и к ожирению.

## РОЛЬ НАСЛЕДСТВЕННЫХ ФАКТОРОВ В РАЗВИТИИ НАРУШЕНИЙ ПИЩЕВОГО ПОВЕДЕНИЯ

Хорошо известно, что такие психические нарушения, как расстройства приема пищи (нервная анорексия, нервная булимия и компульсивное переедание), могут приводить к развитию алиментарного ожирения. Использование близнецовых и семейных методов позволило получить подтверждение роли наследственности в развитии ожирения и нарушений пищевого поведения [61–63]. В частности, было продемонстрировано, что от 47% до 90% индивидуальной вариабельности массы тела можно отнести за счет влияния генетических факторов [64]. Коэффициент наследуемости одного из расстройств пищевого поведения — нервной булимии — составил 55%, а у родственников лиц, страдающих нервной анорексией, шансы развития расстройств приема пищи были повышены в 11,3 раза по сравнению с родственниками лиц из группы контроля [62][65].

В качестве попытки объяснить взаимосвязь наследственности и массы тела была предложена теория поведенческой восприимчивости (behavioural susceptibility theory, BST), согласно которой генетические факторы оказывают влияние на вес путем регуляции аппетита, что проявляется в виде различных паттернов пищевого поведения [66]. Согласно этой теории, ожирение является результатом сочетания генетической наклонности к перееданию и средовых факторов, создающих благоприятные условия для повышенного потребления пищи, что приводит к положительному энергетическому балансу. Показано, что определенные паттерны пищевого поведения ассоциированы с индивидуальными различиями в наборе веса и массе тела и что различия пищевого поведения являются наследуемыми [67][68].

Обнаружены ассоциации с перееданием полиморфных вариантов в генах, продукты которых участвуют в регуляции чувства насыщения (грелин, рецептор меланокортина 4, дофаминовый рецептор D2, белок-переносчик серотонина и др.), а также связь с развитием булимии полиморфных вариантов в генах рецептора эстрогена 1, каннабиноидного рецептора 1, а также в гене FTO [69]. Результаты близнецового исследования с использованием полигенных шкал риска (polygenic risk scores — PRS) продемонстрировали, что ассоциация с ожирением PRS для ИМТ была опосредована различными паттернами пищевого поведения (нерегулярным и нездоровым питанием у мужчин и привычкой к перекусам у представителей обоих полов) [70].

Ранее было обнаружено, что экспрессия генов, связанных с ИМТ, повышена в центральной нервной системе (ЦНС), в областях, отвечающих за процессы обучения и консолидации памяти, в частности, гиппокампе [71]. Впоследствии было показано, что для областей мозга, обогащенных генами, ассоциированными с ИМТ, характерны общие транскрипционные сигнатуры, связанные с ожирением [72]. Для пациентов с генетическими дефектами выработки лептина, у которых при рождении наблюдается нормальный вес, затем рано возникает гиперфагия и развиваются морбидное ожирение, сниженный иммунитет и ряд нейроэндокринных расстройств, включающих аменорею и инсулинорезистентность, был характерен тип пищевого поведения, связанный с почти непрерывной потребностью в еде, причем энергетическая ценность потребляемой пищи была почти в пять раз выше, чем у лиц без ожирения [73]. Назначение таким пациентам препарата, являющегося аналогом лептина, было связано с уменьшением чувства голода, увеличением оценки насыщения, снижением энергетической ценности потребляемой пищи, повышением уверенности в себе, и приводило к значительному снижению массы тела после 15 нед применения [74][75].

Следует отметить, что недостаточность лептина связана с развитием депрессии как у человека, так и у модельных объектов [76]. Роль лептина в формировании пищевого поведения включает в себя не только гомеостатический компонент контроля энергетического баланса, но и гедонистический аспект приема пищи [74]. Сообщалось о том, что лептин воздействует на области головного мозга, образующие систему вознаграждения, снижая степень положительной обратной связи за счет подавления дофаминергической передачи нервных импульсов [77]. Другим аспектом влияния лептина на пищевое поведение является его участие в регуляции восприятия сладкого вкуса вкусовыми рецепторами [78].

Результаты нескольких исследований указывают на возможную роль в развитии нарушений пищевого поведения гормонов, секретируемых белой жировой тканью (адипокинов), в частности адипонектина [79–81]. Установлено, что адипонектин действует в аркуатном ядре гипоталамуса, усиливая аппетит путем активации рецептора AdipoR1 и аденозинмонофосфат-активируемой протеинкиназы (AMPK) [82]. Снижение концентрации адипонектина в плазме обнаруживалось у пациентов с инсулинорезистентностью, ожирением, диабетом 2 типа и ишемической болезнью сердца [83][84].

## РОЛЬ КИШЕЧНОГО МИКРОБИОМА В РАЗВИТИИ ОЖИРЕНИЯ

Одним из важных факторов, влияющих на развитие ожирение, является состав микробного сообщества, населяющего желудочно-кишечный тракт человека, исследования которого были начаты еще в прошлом веке [85]. Последние десятилетия ознаменовались быстрым увеличением числа исследований симбионтной роли микроорганизмов в организме человека. Зачастую это приводит к противоречиям в использовании специальных терминов. Как правило, для обозначения таксономического состава микроорганизмов в определяемом образце используют термин «микробиота», для характеристики набора генов, содержащихся в образце, применяют термин «метагеном», а «микробиомом» называют совокупность генов микробного сообщества [86]. Общее количество бактерий, населяющих желудочно-кишечный тракт человека, достигает 1014, превышая общее число клеток организма [87], в то время как количество генов, составляющих кишечный микробиом, превосходит количество генов в геноме человека почти в 1000 раз [88]. В микробном сообществе кишечника человека преобладают пять типов бактерий: Actinobacteria, Bacteroidetes, Firmicutes, Proteobacteria и Verrucomicrobia [89]. К настоящему времени накоплен достаточно большой объем данных о механизмах влияния микробиоты кишечника на развитие ожирения (рис. 2) [86][90–92].

**Figure:**
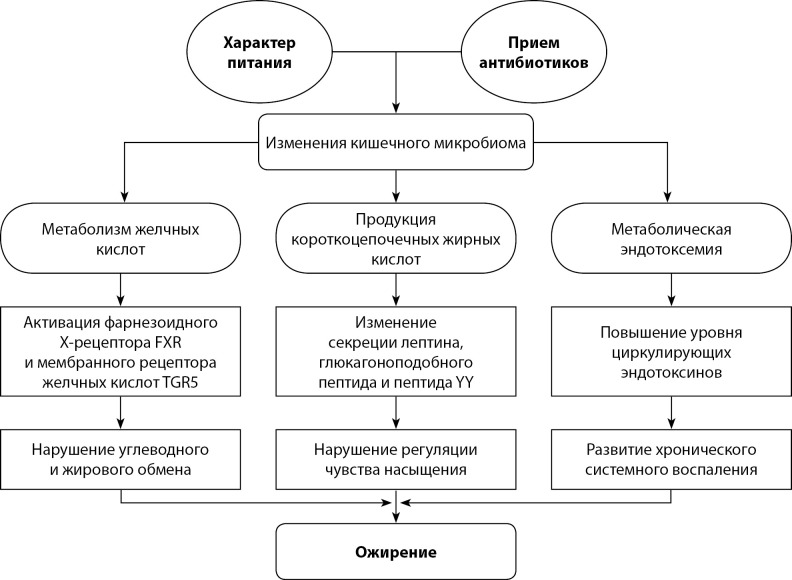
Рисунок 2. Метаболические последствия изменения микробиома кишечника.

Более 15 лет назад было отмечено, что у мышей, которые находились в стандартных лабораторных условиях, количество жировой ткани было намного выше, чем у мышей, содержавшихся в безмикробной среде [93]. Впоследствии были обнаружены значительные различия в бактериальном составе кишечника у мышей с генетической моделью ожирения (ob/ob) по сравнению с мышами без ожирения (ob/+) и мышами дикого типа (+/+), находившимися на одинаковой диете, богатой углеводами; в частности, у мышей с ожирением было относительно снижено число бактерий типа Bacteroidetes и повышено содержание бактерий типа Firmicutes [94]. Перенос микробиома мышей с ожирением стерильным особям приводил к значительному увеличению содержания жира у колонизированных мышей [95].

Для изучения влияния генетических факторов на состав микробиоты человека в основном используется метод анализа ассоциаций. В результате GWAS были выявлены локусы генов, ассоциированные с определенными паттернами кишечной микробиоты [96–98]. Полиморфный локус в гене FUT2 ассоциирован с численностью бактерий Ruminococcus torques семейства Lachnospiraceae класса Clostridia типа Firmicutes, которые специализируются на расщеплении сложных углеводов [99]. Ген FUT2 кодирует фермент альфа-1,2-фукозилтрансферазу, отвечающую за секрецию фукозилированных гликанов слизистой оболочкой желудочно-кишечного тракта. Продемонстрировано, что у лиц, гомозиготных по полиморфному варианту, связанному с появлением стоп-кодона в гене FUT2 (rs601338*A/A), не экспрессируются антигены системы AB0 в клетках слизистой оболочки кишечника, а также уменьшена численность бактерий Ruminococcus torques и Ruminococcus gnavus [100]. В локусе 9q21 обнаружены SNP, ассоциированные с увеличением численности бактерий рода Allisonella семейства Veillonellaceae класса Clostridia типа Firmicutes [100]. Локус 9q21 включает ген RFK, кодирующий фермент рибофлавинкиназу, который катализирует фосфорилирование витамина B2 (рибофлавина), и GCNT1, кодирующий фермент гликозилтрансферазу, участвующую в биосинтезе муцина. Эти ферменты вовлечены в метаболизм бактерий кишечного микробиома и регуляцию иммунной защиты, играя важную роль во взаимодействии организма хозяина и микробиоты кишечника [101]. Полиморфный маркер rs61841503, ассоциированный с численностью бактерий рода Peptostreptococcaceae класса Clostridia типа Firmicutes, расположен в гене CUBN (10p13), кодирующем белок кубилин, который является кишечным рецептором для витамина В12 и требуется для его абсорбции [100]. Витамин В12 необходим для адекватного взаимодействия организма хозяина с микробиомом; в эксперименте in vitro были продемонстрированы существенные изменения микробного состава кишечника после добавления витамина В12 [102]. В локусе 2q36 идентифицирован блок сцепления, включающий 10 полиморфных вариантов, ассоциированных с семейством Peptococcaceae класса Clostridia типа Firmicutes, для которого обнаружена отрицательная связь с уровнями хромогранина А и кальпротектина, являющихся маркерами воспаления в кишечнике [100]. Данный блок локализован в межгенном пространстве рядом с геном субстрата инсулинового рецептора IRS1, связанным с развитием инсулинорезистентности и диабета 2 типа [103]. Продемонстрировано, что так называемая «западная диета», отличающаяся высоким содержанием жиров и углеводов, воздействует на состав микробиома кишечника, способствуя повышенному образованию желчных кислот, в частности, дезоксихолевой кислоты, вызывая активацию сигналинга фарнезоидного Х-рецептора и интерферона I, что приводит к повреждениям клеток Панета, обеспечивающих иммунную защиту тонкого кишечника [104]. Наиболее значимая ассоциация с составом микробиоты была обнаружена для локуса гена лактазы LCT, связанного с повышением содержания Bifidobacterium, отражающим взаимосвязь генетических факторов и характера питания [100].

Повышение числа бактерий типа Firmicutes и снижение содержания бактерий типа Bacteroidetes является характерным признаком микробиоты больных с ожирением. Обратное соотношение наблюдалось после 1 года соблюдения диеты или бариатрической хирургии (шунтирования желудка) [90][105]. У пациентов с ожирением также наблюдается сниженное содержание бактерий типа Bacteroidetes по сравнению с лицами с нормальным ИМТ или пациентами с анорексией, но количество бактерий типа Firmicutes при этом не отличается. Метагеномные исследования близнецовых пар с ожирением и без такового продемонстрировали, что при ожирении снижалось бактериальное разнообразие и содержание Bacteroidetes, но повышалась доля Actinobacteria [92].

Несмотря на активное проведение исследований влияния микробиоты на развитие морбидного ожирения, в настоящее время мало данных о влиянии бариатрической хирургии на микробиом пациентов с ожирением. Оценка состояния микробиоты после бариатрической хирургии (бандажирования или шунтирования желудка по Roux-Y) позволила обнаружить снижение генетического разнообразия, коррелирующее с висцеральным ожирением и наличием осложнений (диабета, артериальной гипертензии), у 75% пациентов [106]. Результаты анализа микробиоты беременных женщин с бариатрическими хирургическими вмешательствами в анамнезе продемонстрировали, что у перенесших мальабсорбтивные операции наблюдалось увеличение числа бактерий Enterococcus и Streptococcus (тип Firmicutes), Escherichia/Shigella (тип Proteobacteria) и Rothia (тип Actinobacteria) и снижение содержания Anaerostipes (тип Firmicutes) [107]. Сообщается также о повышении количества Firmicutes и Proteobacteria у пациентов после шунтирования желудка, в то время как после рукавной гастропластики наблюдалось увеличение числа Bacteroidetes [108]. Отмечается также повышение содержания Lachnospiraceae и Roseburia (тип Firmicutes) у пациентов, достигнувших ремиссии диабета после хирургического вмешательства [108]. Спустя 10 лет после хирургического лечения морбидного ожирения у пациентов было повышено содержание Verrucomicrobiaceae (тип Verrucomicrobia) и Streptococcaceae (тип Firmicutes) и снижено содержание Bacteroidaceae (тип Bacteroidetes) по сравнению с неоперированными пациентами [109]. Относительное повышение содержания Akkermansia muciniphila (тип Verrucomicrobia) коррелировало с ремиссией диабета [109]. У пациентов после шунтирования желудка по Roux-Y было продемонстрировано увеличение содержания протеобактерий Rothia, Aggregatibacter, Granulicatella, Citrobacter, Janthinobacterium и Klebsiella, а также наблюдалось изменение содержания бактерий из рода Firmicutes (повышение числа Streptococcus, Enterococcus, Lactococcus, Veillonella и Granulicatella и снижение Ruminococcus, Blautia и Roseburia) [110]. Изучение микробиоты пациентов с ожирением, в том числе морбидным, и исследование эволюции микробиома после операций бариатрической хирургии представляет значительный интерес для создания методов таргетной терапии.

## ОЖИРЕНИЕ И COVID-19

В настоящее время человечество столкнулось с глобальной угрозой в виде пандемии COVID-19, вызванной новым коронавирусом тяжелого острого респираторного синдрома 2 (severe acute respiratory syndrome coronavirus 2 — SARS-CoV-2). COVID-19 характеризуется высоким уровнем заболеваемости и смертности среди лиц, имеющих сопутствующую патологию, в особенности поражение сердечно-сосудистой системы и ожирение. Подтверждена ассоциация между ожирением (ИМТ ≥35 кг/м2) и повышенным риском осложненного течения и смертности от COVID-19 [111–117]. Результаты анализа генетических факторов риска развития ожирения и COVID-19 с использованием данных GWAS, выполненного в когорте Биобанка Великобритании (UK Biobank), продемонстрировали, что PRS для ИМТ были значимо ассоциированы с риском тяжелого течения коронавирусной инфекции [118]. При проведении GWAS была обнаружена связь с COVID-19, в том числе с осложненным течением, локуса гена AB0, находящегося в блоке сцепления с полиморфными вариантами, ассоциированными с уровнем глюкозы и инсулина в крови, уровнем гликированного гемоглобина, инсулинорезистентностью, диабетом, уровнем липопротеидов низкой плотности и триглицеридов [119][120]. Другой полиморфный вариант, ассоциированный с течением COVID-19, расположен в локусе 5p13.3 в гене рецептора системы натрийуретических пептидов типа С (NPR3) и находится в состоянии неравновесного сцепления с маркерами ожирения, в том числе абдоминального [121][122]. Ранее было продемонстрировано, что ген NPR3 в адипоцитах является мишенью микроРНК miR-146a, которая участвует в регуляции инсулинорезистентности и липогенеза [123]. Использование метода менделевской рандомизации, позволяющего определять причинно-следственные связи, выявило ассоциацию между генетически детерминированным повышением ИМТ и уровня липопротеидов низкой плотности с риском COVID-19 [124]. Таким образом, результаты ассоциативных исследований дают основания полагать, что в основе наблюдаемой корреляции ожирения с риском COVID-19 и неблагоприятными исходами заболевания лежат полиморфные варианты в генах, влияющих на углеводный и липидный обмен.

Известно, что вирус SARS-CoV-2 использует для проникновения в клетки рецептор ACE2, который экспрессирован в подкожной и висцеральной жировой ткани на значительно более высоком уровне, чем в ткани легких [125][126]. Отмечается, что по уровню экспрессии ACE2 в жировых клетках и их предшественниках пациенты с ожирением схожи с индивидуумами с нормальной массой тела, но поскольку у пациентов с ожирением повышено содержание жировой ткани в организме и, соответственно, количество ACE2-продуцирующих клеток, полагают, что это приводит к повышенной выработке ACE2 [126–128].

Одним из предполагаемых рецепторов SARS-CoV-2 является дипептидилпептидаза-4 (DPP4), которая была идентифицирована как один из новых адипокинов жировой ткани [129]. DPP4 представлена на апикальных поверхностях поляризованного эпителия различных органов, таких как легкие и печень, и играет важную роль в гомеостазе глюкозы, воспалении и иммунной системе [130][131]. Продемонстрировано, что повышение уровня DPP4 приводит к хронической субклинической активации иммунной системы и персистирующему воспалению [130]. Активация DPP4 отмечена при ожирении, особенно при развитии инсулинорезистентности, а ингибирование DPP4 предотвращало развитие фиброза белой жировой ткани у мышей с ожирением [132][133]. Клетки жировой ткани, предположительно, являются одним из основных источников циркулирующей DPP4; в частности, были продемонстрированы секреция DPP4 клетками жировой ткани и повышение уровня циркулирующей DPP4 у пациентов с ожирением по сравнению с индивидуумами с нормальной массой тела [129][134]. Повышенная экспрессия DPP4 в жировой ткани пациентов с ожирением может способствовать проникновению SARS-CoV-2 в клетки. Интересно, что ингибиторы DPP4 (глиптины) предложены в качестве препаратов 2–4-го ряда для лечения диабета 2-го типа и могут использоваться в комбинированной терапии COVID-19 [135].

CD147, предполагаемый альтернативный рецептор для SARS-CoV-2, положительно коррелирует с ИМТ, возможно, влияя на заболеваемость и осложненное течение COVID-19 у пациентов с ожирением [128]. Протеаза TMPRSS2, необходимая для праймирования шиповидного белка (S-белка) вируса SARS-CoV-2, экспрессируется в жировой ткани, хотя и на низком уровне, в то время как протеаза фурин, обеспечивающая активацию вируса путем расщепления S-белка, экспрессируется в жировой ткани при ожирении, а также во время адипогенеза [136][137]. Фурин обеспечивает проникновение SARS-CoV-2 в клетки, а за счет праймирования S-белка способствует выходу из клеток вирусных частиц, которые могут атаковать соседние клетки или попадать в кровоток [138].

Избыточное содержание жира приводит к превышению возможностей клеток жировой ткани по его хранению, вследствие чего при биопсии жировой ткани тучных людей с инсулинорезистентностью обнаруживается увеличение количества погибших и отмирающих адипоцитов, сопровождающееся скоплением инфильтрирующих макрофагов [139]. Активированные макрофаги способствуют развитию системного воспаления, характеризующегося увеличением циркулирующих уровней цитокинов, таких как фактор некроза опухоли альфа (TNF), интерлейкины-6 (IL6) и 1-бета (IL1b) [140]. Системное хроническое воспаление является патогенетической точкой соприкосновения, предрасполагающей к развитию тяжелых осложнений у пациентов с ожирением при COVID-19 [141]. Быстрое ухудшение состояния у пациентов с COVID-19 связано с провоспалительным цитокиновым штормом, характеризующимся увеличением системного уровня TNF, IL6, IL2 и IL7, гранулоцитарного колониестимулирующего фактора, хемокинов CXCL10 и CCL2 [142].

Продемонстрировано, что морбидное ожирение приводит к нарушению почти всех функций и свойств стромальных/стволовых клеток жировой ткани (ASC) и способствует переключению нормальной регуляторной активности на прогипоксическую и провоспалительную [143][144]. При ожирении ASC и мезенхимальные стволовые клетки (MSC) теряют свои основные свойства, что сопровождается нарушением их мультипотентного состояния из-за снижения экспрессии генов основных факторов плюрипотентности — октамерсвязывающего белка 4 (OCT4), фактора транскрипции SRY-Box 2 (SOX2), фактора транскрипции гомеобокса Nanog (NANOG), гомолога экзонуклеазы-1 РНК (REX1) и гомеобоксного белка hox-C10 (HOXC10) [145][146]. У пациентов с ожирением наблюдается снижение дифференцировки эндотелиальных клеток, о чем свидетельствует снижение секреции фактора роста эндотелия сосудов A (VEGF), фактора роста гепатоцитов (HGF), фактора роста фибробластов 2 (FGF2) и тромбоцитарного фактора роста (PDGF) [147][148]; изменение иммуномодулирующей способности, что проявляется ингибированием пролиферации лимфоцитов, рекрутированием воспалительных моноцитов и поляризацией макрофагов M1 [149]; изменение внутриклеточного метаболизма, проявляющееся в увеличении продукции активных форм кислорода, нарушении функции митохондрий и подавлении сиртуина 1–6 (SIRT1–6) [143][150][151].

Как уже отмечалось, при ожирении могут возникать повреждения первичных ресничек, в том числе в ASC, что нарушает их уникальную сигнальную функцию. Цитокины IL6 и TNF, ассоциированные с ожирением, также способны индуцировать дефекты цилиогенеза ASC [144][145][152]. ASC с поврежденными первичными ресничками не способны выполнять свои физиологические функции и вместо этого начинают оказывать провоспалительное действие, секретируя воспалительные цитокины, такие как IL6 и IL8 [144][152].

Продемонстрирована связь нарушений функций ASC/MSC с развитием легочного фиброза [153–155]. У пациентов с COVID-19, страдающих ожирением, могут развиваться более тяжелые поражения легких с легочным фиброзом вследствие нарушения функций MSC, индуцированного ожирением [131]. Обсуждается возможность использования ASC/MSC в качестве клеточной терапии легочного фиброза и бронхолегочной дисплазии за счет регулирования популяций иммунных клеток в легких, модуляции дифференцировки клеток, в частности фибробластов, и секретирования различных факторов роста [156][157]. Эти клетки могут также быть использованы для терапии инфекционных заболеваний из-за их иммуносупрессивных или иммуномодулирующих свойств в зависимости от продукции цитокинов и контекста иммунного ответа [158]. Продолжаются клинические испытания, направленные на оценку эффективности и безопасности ASC/MSC для регулирования иммунной системы, предварительные результаты которых дают основания для оптимизма в отношении использования ASC/MSC в качестве регуляторов пролиферации Т-клеток и взаимодействия с патогенами [159][160]. Эти клетки обладают антимикробным потенциалом [101], что подчеркивает значимость ASC/MSC в патогенезе инфекционных заболеваний, а также возможность их применения в качестве новой клеточной терапии для пациентов с COVID-19 с ожирением.

## ЗАКЛЮЧЕНИЕ

В результате проведенных клинико-генетических исследований к настоящему времени накоплен значительный объем данных о синдромальных и несиндромальных формах ожирения. Получено подтверждение участия генов, кодирующих компоненты лептин-меланокортинового пути, в развитии моногенных и полигенных форм ожирения. Имеются сведения о вовлеченности кишечного микробиома в развитие ожирения и его эволюции после бариатрических вмешательств. С учетом вышеизложенного, дальнейшее изучение проблемы ожирения должно быть направлено на выявление взаимосвязей на уровне патогенетических молекулярных механизмов с применением системного комплексного подхода, сочетающего методологию оценки комплекса психологических, клинико-инструментальных, биохимических параметров, вклада генетических факторов и состояния микробиоты. Это позволит идентифицировать потенциальные мишени для лекарственной терапии и создаст предпосылки для разработки персонализированных эффективных подходов в диагностике, лечении и профилактике ожирения.
